# A novel system to analyze oral frailty of mice

**DOI:** 10.18632/aging.204568

**Published:** 2023-03-29

**Authors:** Tetsuya Goto, Eriko Kuramoto, Ayano Kitawaki

**Affiliations:** 1 Department of Oral Anatomy and Cell Biology, Graduate School of Medical and Dental Sciences, Kagoshima University, Kagoshima 890-8544, Japan

**Keywords:** trigeminal nuclei, occlusal force, mastication, electromyogram, pathology of Alzheimer’s disease

Oral frailty has been recently proposed as a novel frailty phenotype and defined as a decrease in oral function coexisting with a decline in cognitive and physical functions [1]. Oral frailty is currently attracting attention, especially in relation to cognitive function [2]. Recent clinical studies have found that patients with Alzheimer’s disease (AD) have impaired masticatory function from an early stage of mild cognitive impairment (MCI) [3].

The histopathological analysis is essential to clarify whether oral frailty with cognitive decline is caused by abnormalities in the masticatory muscles, such as sarcopenia, or abnormalities in the trigeminal nuclei, which is essential for controlling mastication, or both. It is, however, impossible to perform highly invasive histopathological analysis in humans in the MCI stage. Further, there are many variations in the causes of MCI, including the early stage of Alzheimer’s disease, Parkinson’s disease, dementia with Lewy bodies, vascular dementia, frontotemporal dementia [4], making it even more difficult to investigate the etiology of oral frailty. Therefore, it is practical to elucidate the pathological mechanism of oral frailty using mouse models of dementia.

To evaluate oral frailty in humans, a number of methods have been proposed to examine dental status and oral function. For example, evaluation items for the dental states include the number of natural teeth and functioning teeth, tongue thickness as a marker of oral nutrient status, and turbidity of mouth-rinsed water as a marker of oral hygiene. Evaluation items for oral function include maximum occlusal force, chewing ability as a marker of general masticatory performance, maximum tongue pressure, repetitive saliva-swallowing test, tongue-lip motor function test (oral diadochokinesis) with three different sounds (“pa,” “ta,” and “ka”), and oral wettability [6]. However, it is difficult to apply these methods to mice without modification. In mice, oral function is particularly difficult to assess, with only a few previous studies measuring maximum bite force under restraint stress conditions [7]. In order to evaluate the natural oral function of mice, it is best to evaluate the bite during food chewing.

We recently reported a novel system to simultaneously record bite force and electromyogram (EMG) of the masseter muscle in mice, and a new analysis, correlative electro- and force myograph (CLEF) analysis (Figure 1) [5]. This system and CLEF analysis allow for highly accurate estimation of bite force and energy during food mastication in mice, i.e., quantitative evaluation of masticatory function in mice. Evaluation of masticatory function in triple transgenic AD model (3×Tg-AD) male mice with human APP_Swe_, PS1_M146V_, and Tau_P301L_ gene mutations [8] by the CLEF analysis showed less bite force and energy when eating sunflower seeds compared to control mice, C57BL/6J mice. Further, EMG analysis revealed that the masticatory rhythm was delayed in 3×Tg-AD mice. Immunohistochemistry of the trigeminal nuclei revealed that the most intense amyloid β and phosphorylated-tau immunoreactivities were present in the mesencephalic trigeminal nucleus (Vmes) among the trigeminal nuclei of 3×Tg-AD mice at 3 months of age. In addition, a decrease in the number of axons projecting from the Vmes to the trigeminal motor nucleus (Vmo) was found, while no histological changes were observed in the masseter muscle. The Vmes receives sensory input from the periodontal ligament and the masseter muscle spindle and transmits the sensory information to the Vmo, which innervates the masticatory muscles and has the important function of regulating masticatory movements. Altogether, AD pathology in the Vmes of 3×Tg-AD mice might cause the impaired masticatory function. Therefore, it is concluded that the decreased masticatory function observed in 3–4-month-old 3×Tg-AD mice is due to AD pathology in the Vmes and the Vmo. Our novel quantitative analyses of masticatory function using a mouse model of AD enabled a comprehensive understanding of oral frailty pathogenesis. This CLEF analysis can be widely applied not only to the analysis of the causal relationship between AD pathology and masticatory function but also to the evaluation of sarcopenia and the decline of oral function due to aging.

**Figure 1:**
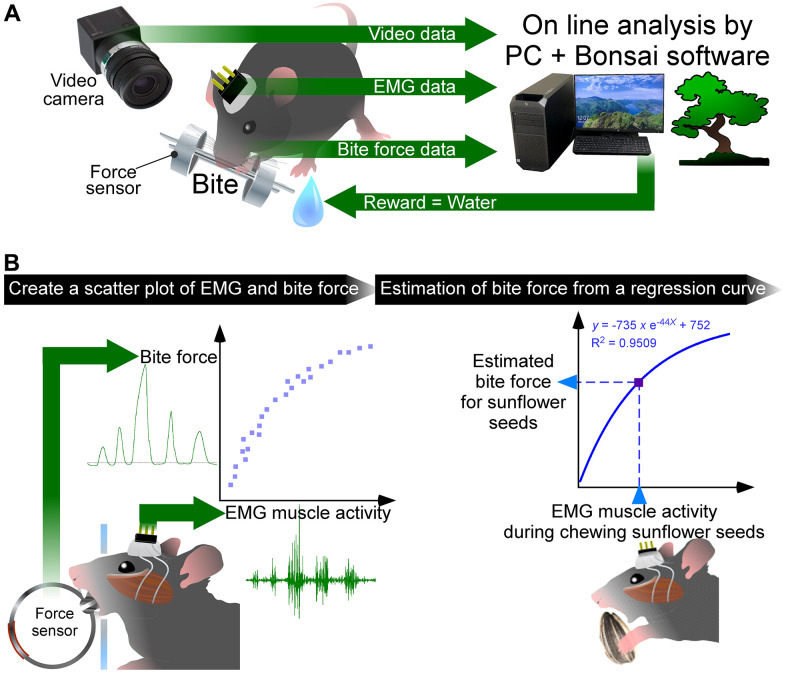
**Newly developed closed-loop system and correlative electro- and force myograph (CLEF) analysis.** (**A**) A closed-loop system to measure the bite force and EMG, simultaneously. The video data, EMG data, and bite force data were stored on a personal computer (PC) with Bonsai software. The bite force values were processed online in Bonsai; when the bite force values exceeded the threshold value, the PC output a signal to reward the mouse with water. (**B**) First, we created a scatter plot showing the correlation between EMG muscle activity and bite force when a mouse chewed a bite force sensor. A non-linear regression curve was created from the scatter plot. Then, the bite force during the mouse chewing sunflower seeds was estimated from EMG muscle activity based on the regression curve. For more details, see text and Kuramoto et al., 2022 [5].

## References

[1] Dibello V, et al. Neural Regen Res. 2021; 16:2149–53. 10.4103/1673-5374.310672 33818486 PMC8354109

[2] Nakamura M, et al. J Clin Med. 2021; 10:1626. 10.3390/jcm10081626 33921265 PMC8068799

[3] Kim MS, et al. Medicine (Baltimore). 2020; 99:e20653. 10.1097/MD.0000000000020653 32502052 PMC7306381

[4] Luis CA, et al. Neurology. 2003; 61:438–44. 10.1212/01.wnl.0000080366.90234.7f 12939414

[5] Kuramoto E, et al. Front Aging Neurosci. 2022; 14:935033. 10.3389/fnagi.2022.935033 35983379 PMC9380890

[6] Tanaka T, et al. J Gerontol A Biol Sci Med Sci. 2018; 73:1661–7. 10.1093/gerona/glx225 29161342

[7] Kim HB, et al. J Dent Res. 2021; 100:960–7. 10.1177/00220345211000263 33719684

[8] Oddo S, et al. Neurobiol Aging. 2003; 24:1063–70. 10.1016/j.neurobiolaging.2003.08.012 14643377

